# Immunological characterization of a long-lasting response in a patient with metastatic triple-negative breast cancer treated with PD-1 and LAG-3 blockade

**DOI:** 10.1038/s41598-024-54041-9

**Published:** 2024-02-09

**Authors:** Licia Rivoltini, Chiara Camisaschi, Giovanni Fucà, Biagio Paolini, Barbara Vergani, Valeria Beretta, Silvia Damian, Matteo Duca, Sara Cresta, Michele Magni, Biagio Eugenio Leone, Chiara Castelli, Filippo de Braud, Francesca De Santis, Massimo Di Nicola

**Affiliations:** 1 Unit of Immunotherapy of Human Tumors, Fondazione IRCCS Istituto Nazionale dei Tumori, Milan, Italy; 2 Immunotherapy and Innovative Therapeutics Unit, Medical Oncology and Hematology Department, Fondazione IRCCS Istituto Nazionale dei Tumori, Via Giacomo Venezian,1, 20133 Milan, Italy; 3 Biomarkers Unit, Department of Applied Research and Technical Development, Fondazione IRCCS Istituto Nazionale dei Tumori, Milan, Italy; 4 Pathology A Unit, Department of Pathology, Fondazione IRCCS Istituto Nazionale dei Tumori, Milan, Italy; 5 Department of Medicine and Surgery, University of Milano-Bicocca, Monza, Italy; 6 Oncology and Hemato-Oncology Department, University of Milan, Milan, Italy

**Keywords:** Cancer, Immunology, Biomarkers

## Abstract

In patients with advanced triple-negative breast cancer (TNBC), translational research efforts are needed to improve the clinical efficacy of immunotherapy with checkpoint inhibitors. Here, we report on the immunological characterization of an exceptional, long-lasting, tumor complete response in a patient with metastatic TNBC treated with dual PD-1 and LAG-3 blockade within the phase I/II study CLAG525X2101C (NCT02460224) The pre-treatment tumor biopsy revealed the presence of a CD3^+^ and CD8^+^ cell infiltrate, with few PD1^+^ cells, rare CD4^+^ cells, and an absence of both NK cells and LAG3 expression. Conversely, tumor cells exhibited positive staining for the three primary LAG-3 ligands (HLA-DR, FGL-1, and galectin-3), while being negative for PD-L1. In peripheral blood, baseline expression of LAG-3 and PD-1 was observed in circulating immune cells. Following treatment initiation, there was a rapid increase in proliferating granzyme-B^+^ NK and T cells, including CD4^+^ T cells, alongside a reduction in myeloid-derived suppressor cells. The role of LAG-3 expression on circulating NK cells, as well as the expression of LAG-3 ligands on tumor cells and the early modulation of circulating cytotoxic CD4^+^ T cells warrant further investigation as exploitable predictive biomarkers for dual PD-1 and LAG-3 blockade.

**Trial registration:** NCT02460224. Registered 02/06/2015.

## Introduction

The clinical studies evaluating anti-PD-1/PD-L1 monotherapy in triple-negative breast cancer (TNBC) have demonstrated a relatively modest level of activity compared to other histologies such as melanoma and non-small cell lung cancer NSCLC^1^. This limited efficacy of PD-1/PD-L1 inhibitors alone may be attributed to the involvement of additional immune checkpoints in the process of cancer immunoediting. Consequently, there is growing interest in investigating combination approaches that target different and complementary immune checkpoints^2^. Among these novel targets, the Lymphocyte Activation Gene-3 (LAG-3) stands out as a particularly promising surface molecule expressed on activated T and NK cells, which exerts negative regulation on immune effector signaling upon ligand engagement^3^. In this report, we present a remarkable and durable complete response to dual PD-1/LAG-3 blockade observed in a patient with metastatic TNBC who participated in the phase I/II study CLAG525X2101C (NCT02460224)^4^.

## Results

### Dynamics of response to dual PD-1/LAG-3 blockade

**Figure 1:**
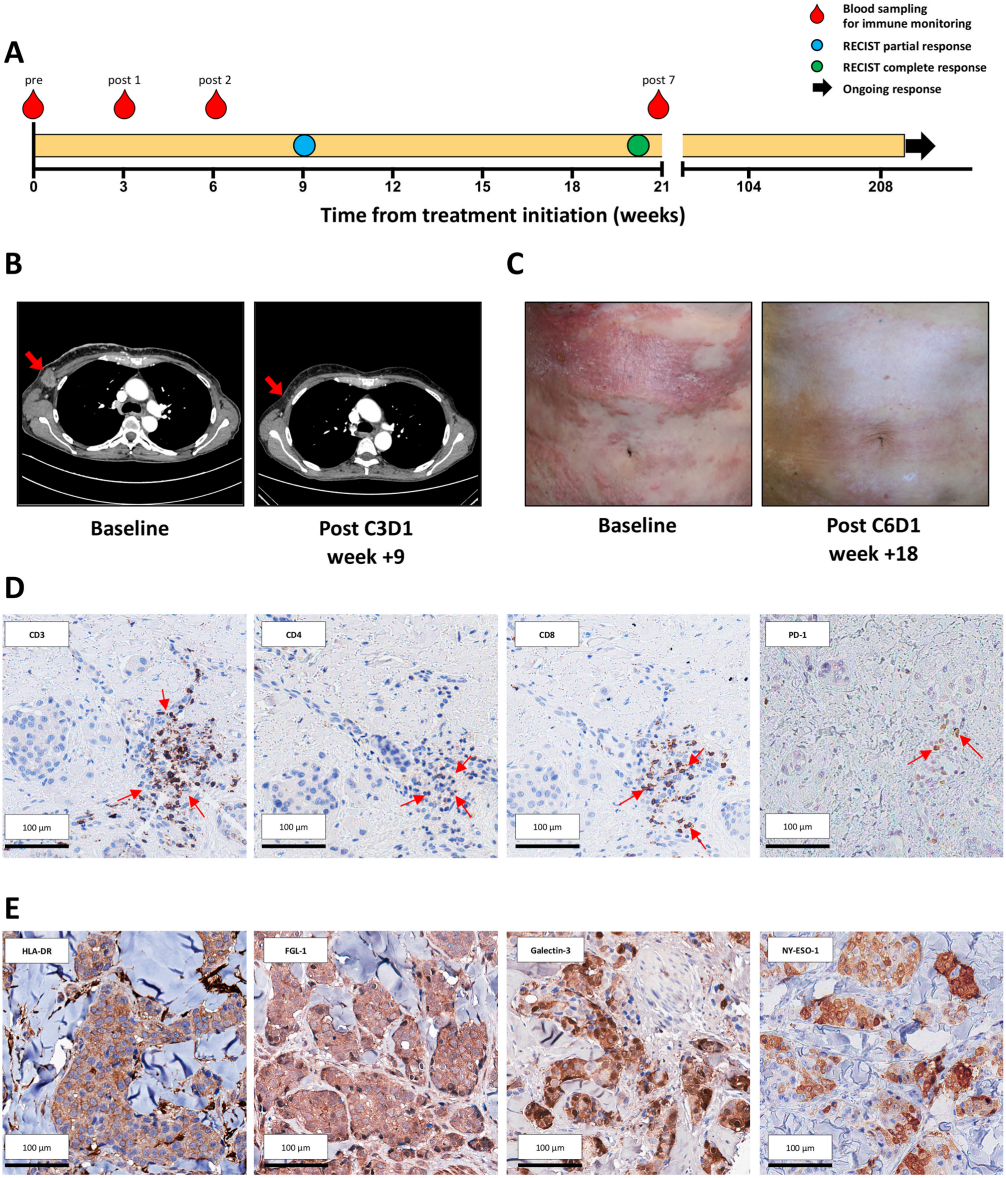
Dynamics of response to dual PD-1/LAG-3 blockade and immune-molecular tumor characterization. (**A**) Shows the timeline of response dynamics together with the time of blood sampling for the immune monitoring. A rapid antitumor activity with a radiological partial response (as illustrated by the computed tomography images of a lymph node metastases in **B**) was achieved at week + 9 and was paralleled by the regression of most of the skin involvement. A complete clinical response of the extensive skin localization was observed at week + 18 (**C**). A complete radiological response (as per RECIST v.1.1) was achieved at week + 20. The immunohistochemical characterization of the tumor microenvironment showed a zonal CD3^+^ infiltrate mainly composed of CD8^+^ cells with rare CD4^+^ and rare PD-1^+^ cells (**D**, images at 20 × magnification). Tumor cells stained positive for the three main known LAG-3 ligands (HLA-DR, FGL-1 and galectin-3) and for the cancer testis antigen NY-ESO-1 (**E**, images at 20 × magnification).

The patient in their 60 s was affected by heavily-pretreated metastatic TNBC with significant skin involvement and lymph nodal metastases. Figure 1A illustrates the dynamics of the clinical and radiological response to the dual PD-1/LAG-3 blockade. After three cycles of the experimental treatment, the first radiological tumor reassessment at week + 9 revealed a near-complete response (CR) of the lymph nodal metastases (Fig. 1B). This response was accompanied by regression in most of the sites of skin involvement. Supplemental Figure S1 displays H&E-stained tissue sections of tumor biopsy specimens (A) and the pathological complete response observed at week + 9 (B). At week + 18, a complete clinical response was achieved in the cutaneous lesions (Fig. 1C), and a RECIST CR was attained at week + 20. As of the data cutoff date for this report, the patient remains on treatment and has remained disease-free during a follow-up period of over 50 months.

### Immune and molecular tumor characterization

**Figure 2:**
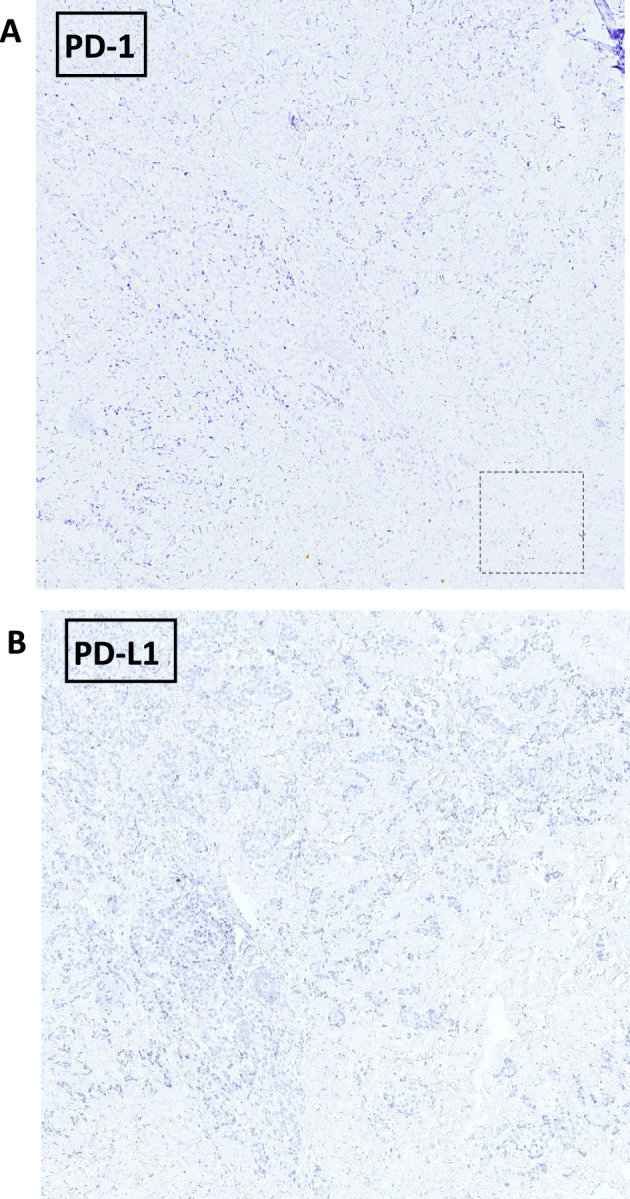
Immunohistochemical staining at 4 × magnification. Immunohistochemical staining for PD-1 (**A**) and PD-L1 (**B**) at 4 × magnification. The dashed line rectangle indicates the area showed at 20 × magnification in Fig. 1D.

**Figure 3:**
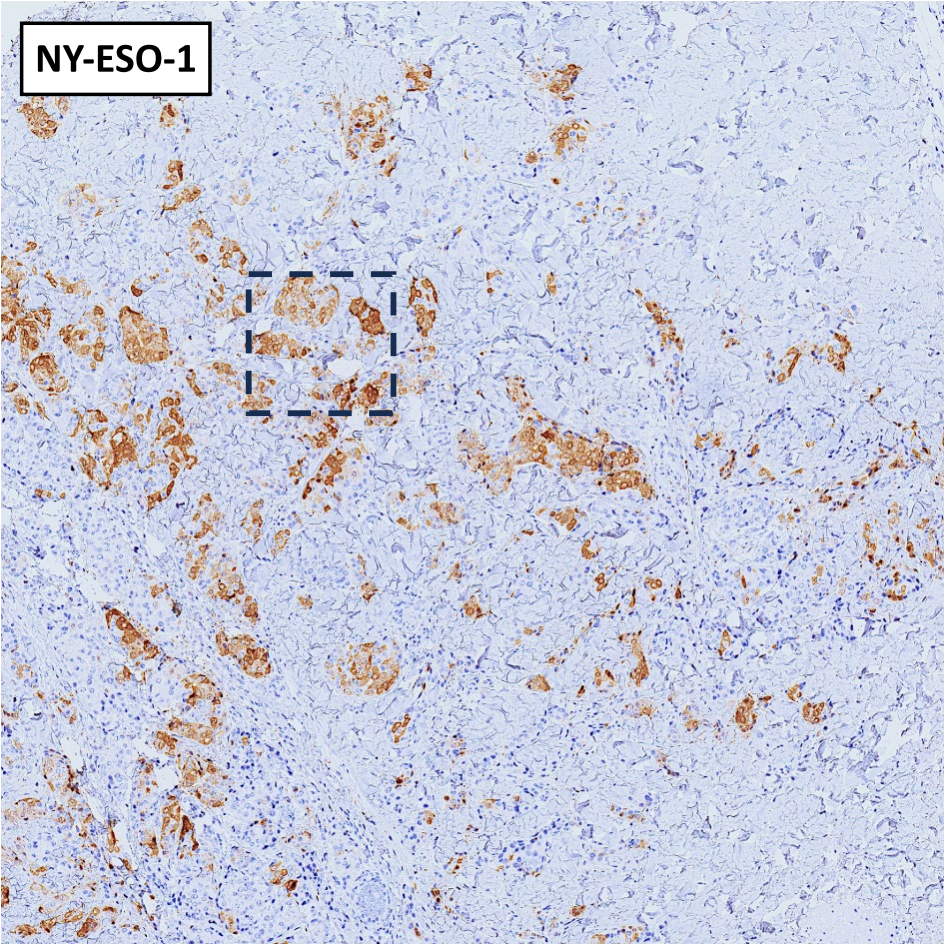
Immunohistochemical staining for NY-ESO-1 at 4 × magnification. The dashed line rectangle indicates the area showed at 20 × magnification in Fig. 1E.

To investigate the basis of this exceptional response, we conducted a characterization of the tumor's molecular and immunological profile. The tumor punch biopsy obtained at baseline from the skin localization on cycle 1 day 1 (C1D1, prior to treatment initiation) revealed a zonal distribution of tumor-infiltrating lymphocytes (TILs) (Supplemental Fig. S1A), with a CD3 + T cell infiltrate ranging from 5 to 30% (Fig. 1D and Supplemental Fig. S2). This range was similar to the median of 20% TILs reported in the literature for TNBC^5^. TIL characterization showed a moderate presence of CD8^+^ T cells with rare CD4^+^ T cells and no detectable infiltration of NK and NKT cellular elements, as demonstrated by the absence of CD56 immunostaining (Fig. 1D). In terms of immune checkpoints, PD-1 expression was scantly and detectable only within the CD8^+^ T cell subset (Figs. 1D and 2A) while LAG-3 was absent in T and any other infiltrating immune cells (Supplemental Fig. S2E). It is worth mentioning that no expression of PD-L1 could be observed (Fig. 2B), whereas three (HLA-DR, FGL-1, and galectin-3) of the multiple ligands for LAG-3^3^ were highly expressed in both cancer and stromal cells (Fig. 1E and Supplemental Fig. S3A–C). The antigenic analysis of tumor cells revealed a microsatellite stable genotype (MSS +), indicating the absence of DNA hyper-mutability underlying the potential immunogenicity of the tumor through the expression of neoantigens^6^. However, the cancer testis antigen NY-ESO-1, which is typically well-represented in TNBC^7^, showed high positivity at tumor level (Figs. 1E, 3).

A tissue biopsy obtained from the responding tumor lesion, nine weeks after treatment initiation, showed no residual cancer cells confirming the complete regression, along with the presence of abundant stroma and histiocytes as detected by histological analysis (Supplemental Fig. S1B); however, no immune infiltrate could be observed, likely due to the late time point of the sampling.

**Figure 4:**
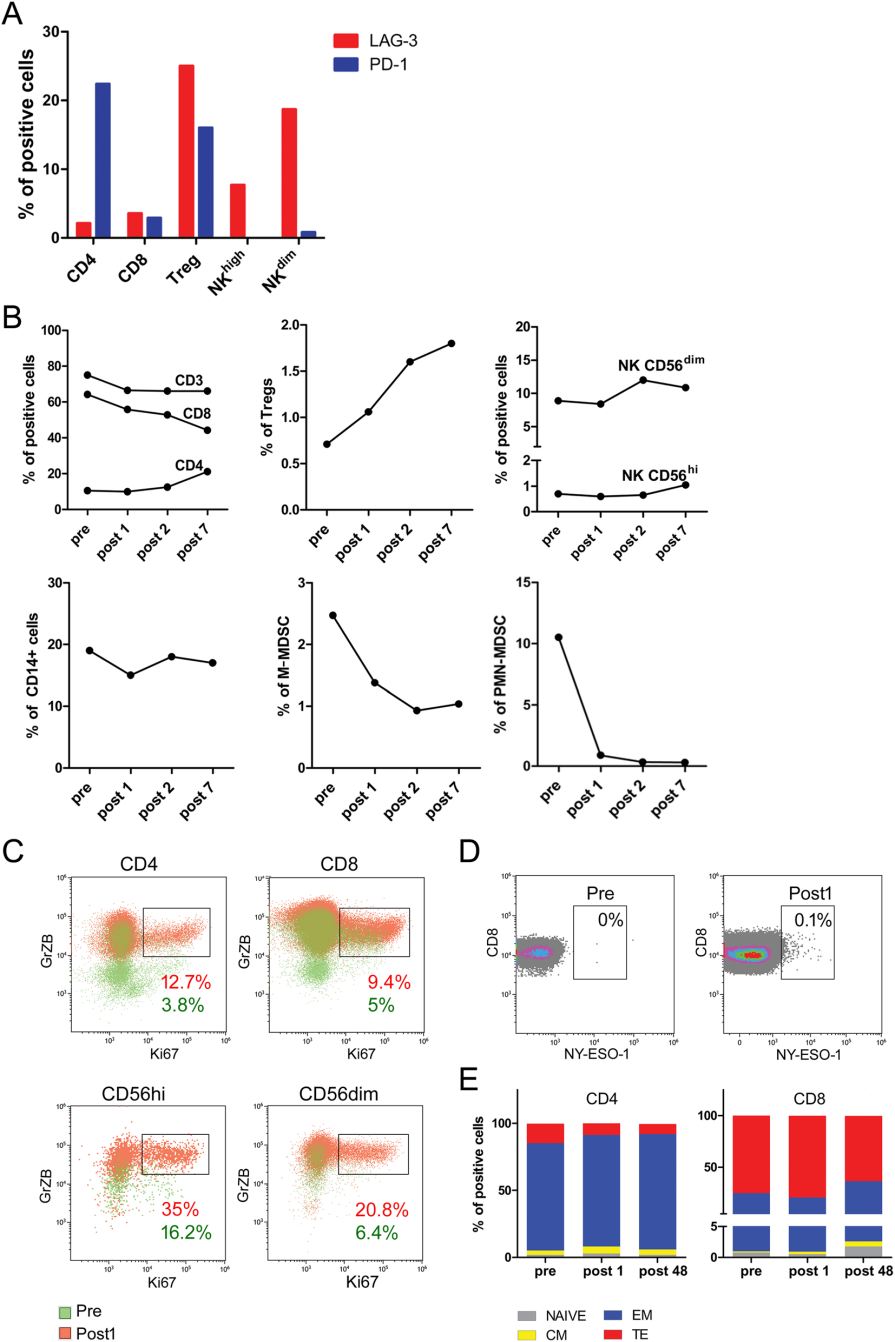
Effects of PD-1/LAG-3 dual blockade on blood immune cell frequency and induction of a lymphocyte-mediated antitumor response soon after treatment initiation. Freshly thawed PBMCs were analysed by flow cytometry. (**A**) Percentage of LAG-3 (red) and PD-1 (blue) positive cells at the baseline in CD4^+^, CD8^+^, Treg (CD4^+^CD25^+^Foxp3^+^) and NK cells (CD16^−^CD56^hi^ and CD16^+^CD56^dim^) are reported in graph. (**B**) Frequency of lymphoid (CD3^+^, CD4^+^, CD8^+^, Treg, NK cells) and myeloid (CD14^+^ monocytes, M-MDSC and PMN-MDSC) populations at the baseline (pre) and after treatments (post 1,2, 7) are shown. (**C)** overlay dot plots show the increased proliferation of GrZB^+^ cells (rectangle gate) after the first treatment (red) compared to the baseline (green) in CD4^+^ and CD8^+^ T cells (upper row) and in NK CD56^hi^ and NK CD56^dim^ (lower row). The frequency of cells expressing both the intracellular markers, Ki67 and GrZB, are reported in the graph as percentages (red = first treatment, green = baseline). (**D**) The presence of NY-ESO-1 specific CD8^+^ T cells was evaluated comparing PBMCs at the baseline and after the first treatment using HLA-A*0201/NY-ESO-1 multimer staining. The percentage of CD8^+^ multimer positive cells was calculated in the CD8^+^CD19^−^ gate and reported in the figure. (**E**) PMBCs were stained with a combination of mAbs to define naïve and memory T cell subsets. Histograms show the percentage of naïve (CD45RA^+^CCR7^+^, grey), effector memory (EM, CD45RA^−^CCR7^−^, blue), central memory (CM, CD45RA^−^CCR7^+^, yellow) and terminally effector (TE, CD45RA^+^CCR7^−^, red) cells in CD4^+^ and CD8^+^ lymphocytes at the baseline (pre), after the first treatment (post 1) and late in the treatment (post 48).

By profiling peripheral blood mononuclear cells (PBMC), we observed that PD-1 was expressed in a significant proportion of CD4^+^ T and Treg cells (23% and 17%, respectively), while poorly represented in CD8^+^ T cells (3%); no detection of this immune checkpoint could instead be noticed in either CD56^dim^ cytotoxic NK or CD56^high^ cytokine-producer NK cell subsets (Fig. 4A). In contrast, LAG-3 was abundantly present in a remarkable fraction of Tregs (26%), a subset of CD8^+^ T (4%) and both the CD56^high^ and CD56^dim^ NK cell sub-populations (8% and 19% respectively) (Fig. 4A).

The dynamic immune monitoring of PBMC revealed that after the first infusion of the combo-therapy (C1D1) and throughout the treatment (up to the 7th cycle), the number of circulating T and NK cells did not significantly change, while circulating Tregs (defined as CD3^+^CD4^+^CD25^+^Foxp3^+^ cells) showed a progressive boost, which is consistent with the occurrence of full-fledged immune-stimulation^8^ (Fig. 4B). Concomitantly, treatment was associated with a strong and rapid decrease in monocytic myeloid-derived suppressor cells (M-MDSCs, CD14^+^HLA-DR^neg^ cells), despite no significant change in the overall CD14^+^ monocytic population, and by a reduction of granulocytic MDSCs (PMN-MDSCs, CD14^−^CD15^+^CD11b^+^ low-density cells), indicating a lowering of the systemic immunosuppression associated with altered myelopoiesis (Fig. 4B). This immune scenario was paralleled by a remarkable increase in the fraction of Ki67 + and granzyme-B double-positive cells in CD4^+^ (3.8% pre *vs* 12.7% post) and CD8^+^ (5% pre *vs* 9.4% post) T lymphocytes, as well as in both the CD56^high^ (16.2% pre *vs* 35% post) and CD56^dim^ (6.4% pre *vs* 20.8% post) NK cell subsets (Fig. 4C), which suggests the ability of PD-1 and LAG3 blockade to unleash proliferating and cytotoxic anti-tumor effectors. This hypothesis is also supported by the evidence that circulating CD8^+^ T cells recognizing the tumor antigen NY-ESO-1, highly expressed on the TNBC cells of this patient (Fig. 1E), exhibited a sustained expansion during treatment as indicated by the staining with HLA-A*0201/NY-ESO-1 peptide pentamers (Fig. 4D). Finally, both CD4^+^ and CD8^+^ T cells showed a significant skew toward an effector-memory (CD45RA-CCR7-) phenotype (Fig. 4E), suggesting a potential ability of the combined PD-1 and LAG-3 inhibition to triggering systemic immunological memory.

## Discussion

In this report, we present the case of a patient with TNBC who experienced a complete tumor regression when treated with a combination of PD-1 and LAG-3 blocking antibodies. In particular this case belongs to a phase I/II, open label, multicenter study investigating the safety and efficacy of the humanized immunoglobulin 4 (IgG4) anti-LAG3 mAb ieramilimab (LAG525) ± the humanized IgG4 anti-PD-1 mAb spartalizumab (PDR001) in patients with advanced solid malignancies^9^.

The analysis of a skin lesion biopsied at baseline revealed a scantly and heterogeneous T cell infiltrate poorly expressing the targeted immune checkpoints PD-1 and LAG3, the absence of PD-L1 expression in both cancer or stromal cells, and a MSS genotype, which are all hallmarks of tumors poorly responding to PD-1/PD-L1 blockade monotherapy, as reported in multiple previous clinical trials^1^. However, the high levels of three (HLA-DR, FGL-1, and galectin-3) of the putative LAG-3 ligands^10^ in cancer cells, confirming the involvement of these molecules in blunting antitumor immunosurveillance^11^, points to the expression of HLA-DR, FGL-1, and/or galectin-3 as potential predictive factor of sensitivity to LAG3 blockade therapy.

As systemic immunity is acknowledged to impact of antitumor immune responses occurring at tumor site^12^, baseline and dynamic immunoprofiling of peripheral blood was performed in the patient here depicted. A prevalent expression of LAG3 with respect to PD-1 was detected in pre-treatment PBMC, particularly in CD8 + T, Treg, and both CD56^dim^ and CD56^high^ NK cells. Based on this evidence, and the high levels of HLA-DR, FGL-1, and galectin-3 cognate ligands at tumor site, it is tempting to speculate that systemic LAG-3 blockade might have triggering an immunological cascade leading to the expansion of active immune effectors which would eventually migrate to tumor site to mediate the complete tumor regression observed in our patient, with PD-1 blocker possibly amplifying and prolonging the immunomodulating effect^13^. Indeed, the administration of LAG3 and PD-1 blocking mAbs was associated with a remarkable increase of multiple activated, cytotoxic and proliferating immune effectors including CD8^+^, CD4^+^ T and NK cells. If the activity of this immunotherapeutic strategy on T cells is expected based on the literature data^10^, we believe a novelty of this report might rely on the remarkable activation and expansion of both cytotoxic and cytokine-producing NK cells observed to occur rapidly upon treatment. These effectors, endowed with an antitumor potential in TNBC patients^14^, have been recently reported to upregulate LAG3 and to acquire mature phenotype as well as effector functions upon LAG3 blockade^15^, thus representing a potentIal mediator of the antitumor activity triggered in vivo by LAG3 inhibition^16^.

The complete tumor regression experienced by this patient was also associated with a substantial modulation of regulatory and suppressive peripheral immune compartments. Notably, the boost of Treg, often paralleling full-fledged immune responses^8^, did not apparently impair the activation of T and NK effectors possibly due to the ability of LAG3 and PD-1 inhibition of making these cells resistant to immunosuppression in vivo^17^. In contrast, M-MDSC and PMN-MDSC rapidly dropped after the first treatment cycle, indicating a direct activity of LAG3/PD-1 blocking on the systemic myeloid population rather than an indirect effect due to the reduced tumor burden. Albeit the molecular mechanisms underlying this process require further investigation, it could be hypothesized that PD-1 expressed on myeloid cells may play a central role in orchestrating immune checkpoint blockade, as recently highlighted^18^; similarly, as Lag3 ligands are abundantly detectable on myeloid cells in different pathological conditions including breast cancer^19^ and soluble LAG3 is key in myeloid cell differentiation^20^, blockade of this checkpoint might have also contributed to myeloid cell reprogramming from immunosuppressive to proinflammatory effectors endowed with antitumor potential^21,22^.

In conclusion, based on the lesson learnt from a single case report, we speculate that the exceptional and long-lasting complete response achieved in this TNBC patient following dual PD-1/LAG-3 blockade may have been driven by a multifaceted immune response, including the reversal of NK cell exhaustion mediated by the LAG-3 axis, the reactivation of cytotoxic and effector-memory T lymphocytes, and the reprogramming or recirculation of myeloid cells. These findings suggest that PD-1/LAG-3 blockade could be an effective treatment approach for tumors expressing the hallmarks of PD-1 blockade resistance but high levels of LAG-3 ligands. Further investigations are needed to confirm whether the tumor and the blood pathways here identified at baseline or early during treatment may represent true predictive biomarkers for anti-LAG-3 blockade-based immunotherapy.

## Methods

### Experimental treatment

In June 2016, the patient started the experimental treatment regimen consisting of LAG525 (a humanized IgG4 anti-LAG-3 monoclonal antibody [mAb]) at a dosage of 240 mg, and spartalizumab (PDR001, a humanized IgG4 anti-PD-1 mAb) at a dosage of 300 mg, administered every three weeks. Tumor response was assessed using RECIST v1.1 criteria in accordance with the study protocol. The data cutoff date for the present report is 01/03/2023.

Formalin-fixed, paraffin-embedded (FFPE) tissue blocks were retrieved, and expert pathologist review was conducted on hematoxylin and eosin (H&E) stained tissue sections. Immunohistochemical characterization utilized the reagents listed in Supplemental Table S1. Antigen retrieval was performed at high temperatures (96–98 °C) using different buffers, and the antigen–antibody reaction was visualized using commercial detection kits (see Supplemental Table S1). Microsatellite instability (MSI) status was determined through polymerase chain reaction (PCR) following international guidelines^5^.

Peripheral blood mononuclear cells (PBMCs) were isolated from blood samples using Ficoll gradient (Leuco-sep polypropylene tubes, Thermo Fisher Scientific) within two hours from withdrawal. The reagents employed for cell staining are provided in Supplemental Table S2. Samples were analyzed using the Gallios Flow Cytometer, and the Kaluza software (Beckman Coulter) was used for data analysis. Positive cells were identified within the live cell population after doublet discrimination, and based on fluorescence minus one (FMO) control.

### Supplementary Information

Supplementary Figure 1.

Supplementary Figure 2.

Supplementary Figure 3.

Supplementary Table 1.

Supplementary Table 2.

## Data Availability

The data generated and/or analyzed during the current study are available from the corresponding author on reasonable request.
