# Supplementary material for: Immunological characterization of a long-lasting response in a patient with metastatic triple-negative breast cancer treated with PD-1 and LAG-3 blockade
Source: Sci Rep. 2024 Feb 9;14:3379. doi: 10.1038/s41598-024-54041-9 (PMC10858221; doi:10.1038/s41598-024-54041-9)

Supplemental Figure 1

Hematoxylin and eosin stained tissue sections of tumor punch biopsies (skin localization) obtained before (A) and 9 weeks after (B) experimental treatment initiation. B) shows a pathological complete response.

A

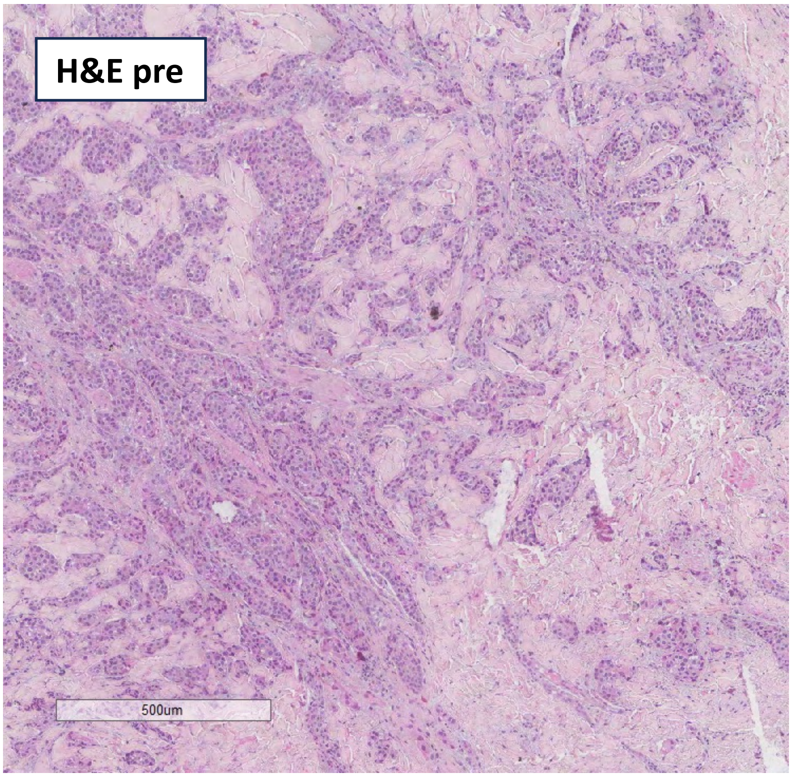

B

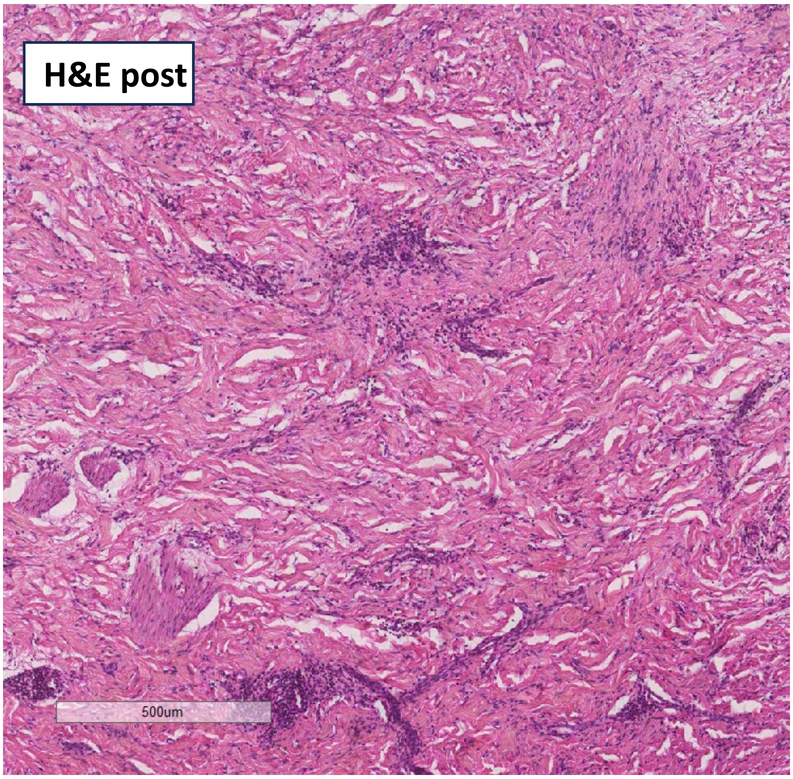

Supplement: Supplementary file 1 — Supplementary Figure 1. [file 41598_2024_54041_MOESM1_ESM.pdf]
