# Supplementary material for: Immunological characterization of a long-lasting response in a patient with metastatic triple-negative breast cancer treated with PD-1 and LAG-3 blockade
Source: Sci Rep. 2024 Feb 9;14:3379. doi: 10.1038/s41598-024-54041-9 (PMC10858221; doi:10.1038/s41598-024-54041-9)

Supplemental Figure 2

Immunohistochemical staining for CD3 (A), CD4 (B), CD8 (C), CD56 (D), LAG3 at 4x magnification. The dashed line rectangle in A) indicates the area showed at 20x magnification in figure 1D.

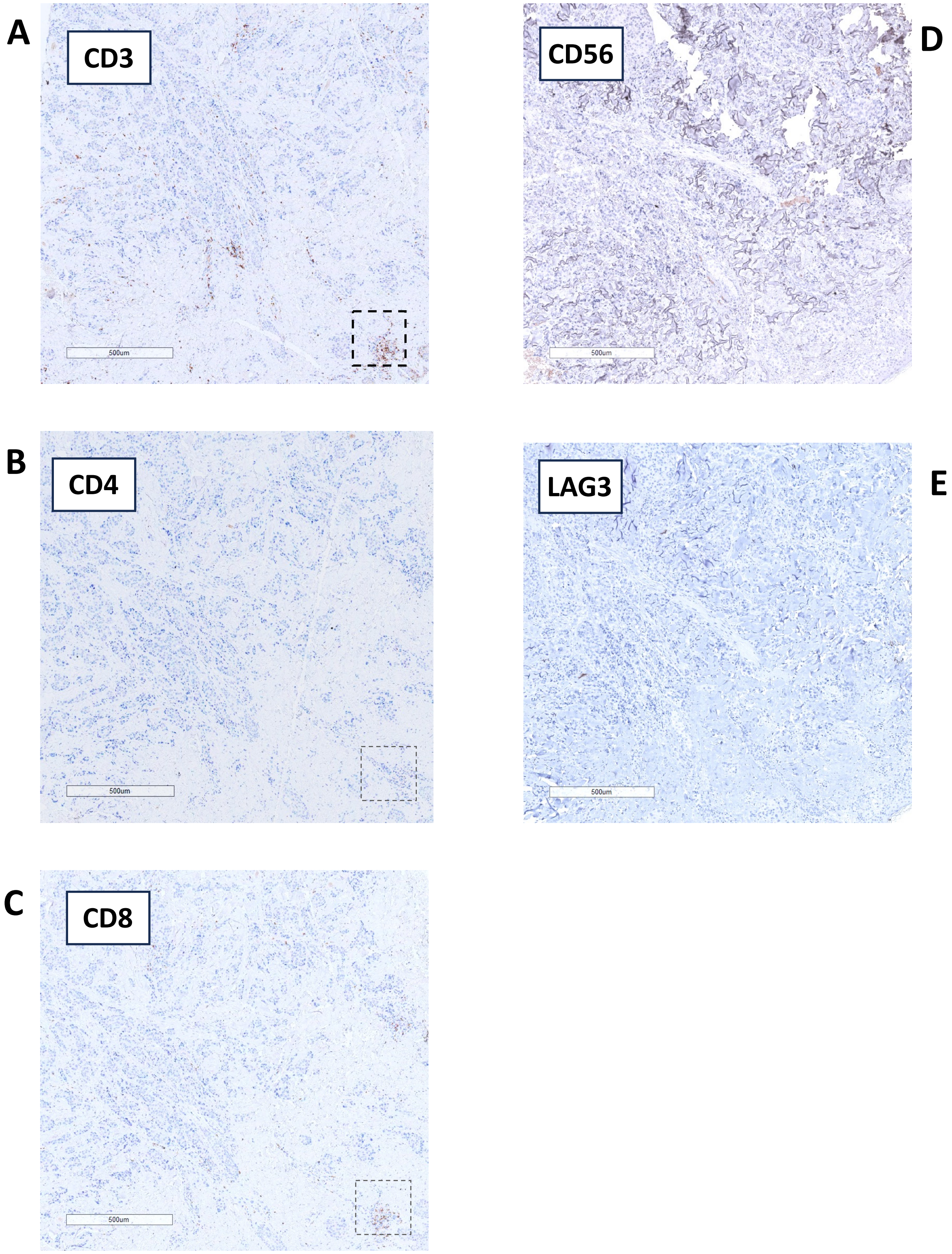

Supplement: Supplementary file 2 — Supplementary Figure 2. [file 41598_2024_54041_MOESM2_ESM.pdf]
