# Supplementary material for: Immunological characterization of a long-lasting response in a patient with metastatic triple-negative breast cancer treated with PD-1 and LAG-3 blockade
Source: Sci Rep. 2024 Feb 9;14:3379. doi: 10.1038/s41598-024-54041-9 (PMC10858221; doi:10.1038/s41598-024-54041-9)

**Supplemental Figure 3**

Immunohistochemical staining for HLA-DR (A), FGL-1 (B), Galectin-3 (C).

The dashed line rectangles indicate the area showed at 20x magnification in figure 1E.

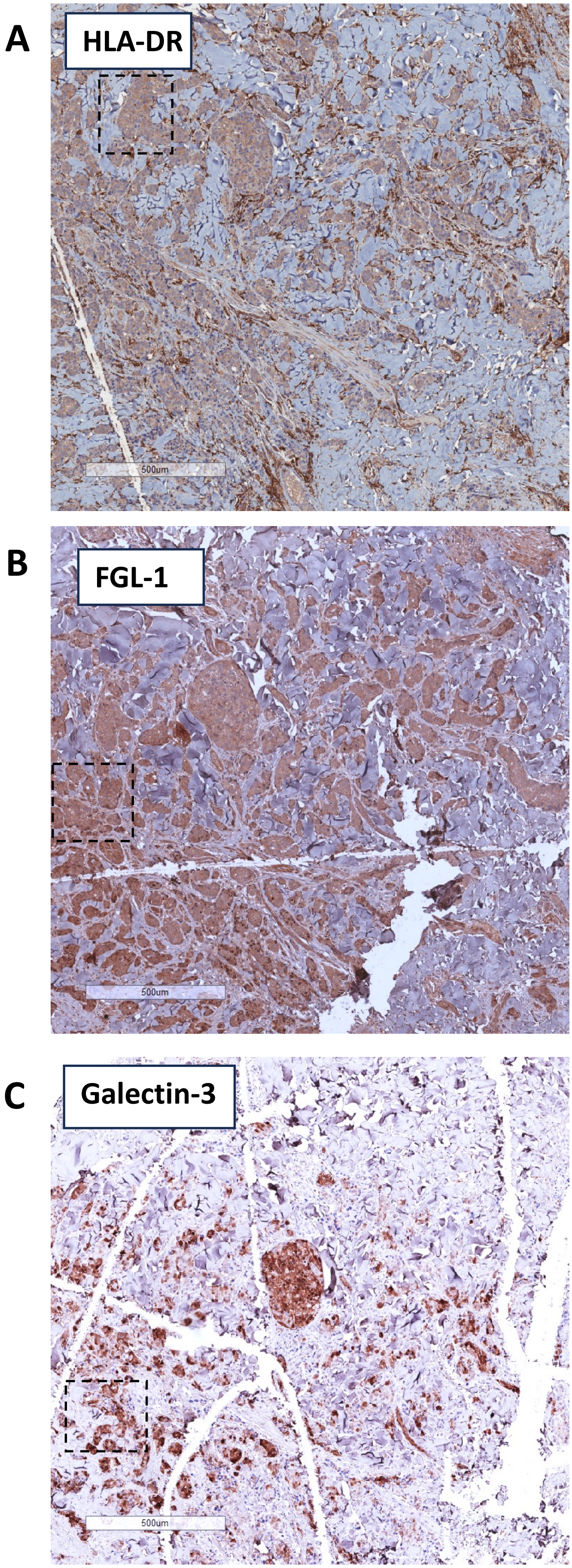

Supplement: Supplementary file 3 — Supplementary Figure 3. [file 41598_2024_54041_MOESM3_ESM.pdf]
