# Supplementary material for: Immunological characterization of a long-lasting response in a patient with metastatic triple-negative breast cancer treated with PD-1 and LAG-3 blockade
Source: Sci Rep. 2024 Feb 9;14:3379. doi: 10.1038/s41598-024-54041-9 (PMC10858221; doi:10.1038/s41598-024-54041-9)
Supplement: Supplementary file 5 — Supplementary Table 2. [file 41598_2024_54041_MOESM5_ESM.pdf]

Supplemental Table 2. Reagents used for cell staining in immune monitoring analyses.

Reagent	Company	Cat#
Anti-hCD3 Percp5.5	BD Biosciences	560835
Anti-hCD3 PE-CF594	BD Biosciences	562310
Anti-hCD4 APC Alexa 750	Beckman Coulter	A94682
Anti-hCD8 Krome Orange	Beckman Coulter	B00067
Anti-hPD-1 PC7	Beckman Coulter	A78885
Anti-hLAG-3 FITC	AdipoGen	AG-20B-0012F
Anti-hCD25 PECF594	BD Biosciences	562403
Anti-hFoxP3 PE	Thermo Fisher Scientific	12-4776-42
Anti-hCD14 APC Alexa 750	Beckman Coulter	A86052
Anti-hCD15 Percp5.5	BD Biosciences	560828
Anti-hCD56 BV510	BD Biosciences Cat	744218
Anti-hHLA-DR FITC	BD Biosciences	555811
Anti-hCD11b PC7	Beckman Coulter	A54822
Anti-hCD16 PE	BD Biosciences	561313
Anti-hCD19 PE	BD Biosciences	555413
Anti-hPDL-1 PE-CF594	BD Biosciences	563742
Anti-hGrZB BV421	BD Biosciences	563389
Anti-hKi67 Alexa700	BD Biosciences	561277
Anti-hCD45RA APC Alexa 750	Beckman Coulter	B49194
Anti-hCCR7(CD197) Alexa 647	BD Biosciences	560816
Pro5® MHC Class I HLA-A*0201/NY-ESO-1 (SLLMWITQV) Pentamer	Proimmune	PeptideCode# 390
eBioscience™ Transcription Factor Fixation/Permeabilization	ThermoFisher	00-5521-00
Live-Dead Fixable Violet Staining Kit	Invitrogen	L34955
