# Supplementary material for: Immunological characterization of a long-lasting response in a patient with metastatic triple-negative breast cancer treated with PD-1 and LAG-3 blockade
Source: Sci Rep. 2024 Feb 9;14:3379. doi: 10.1038/s41598-024-54041-9 (PMC10858221; doi:10.1038/s41598-024-54041-9)
Supplement: Supplementary file 4 — Supplementary Table 1. [file 41598_2024_54041_MOESM4_ESM.pdf]

Supplemental Table 1. Reagents used for immunohistochemistry analyses.

Reagent	Company	Cat#
Anti-hCD3	Dako Agilent	M7254
Anti-hCD4	Dako Agilent	M7310
Anti-hCD8	Dako Agilent	M7103
Anti-NY-ESO-1	Thermo Fisher Scientific	35-6200
Anti-hPD-1	Biocare medical	ACI 3137 AK
Anti-hPD-L1	Dako Agilent	M3653
EnVision FLEX	Dako Agilent	GV800
Anti-hCD56	Dako Agilent	IR628
Anti-hLAG3	Cell Signaling	15372
Anti-hHLA-DR	Thermo Fisher Scientific	MS-133-P1
Anti-hFGL1	Invitrogen- Thermo Fisher Scientific	PA5-97750
Anti-hGalectin-3	Novus Biologicals	NB300-538
UltraVision™ LP Detection System: HRP Polymer	Thermo Fisher Scientific	TL-125-HL
Liquid DAB+ Substrate Chromogen System	Dako Agilent	K3468
